# NF-κB Signaling Pathways in Osteoarthritic Cartilage Destruction

**DOI:** 10.3390/cells8070734

**Published:** 2019-07-17

**Authors:** Moon-Chang Choi, Jiwon Jo, Jonggwan Park, Hee Kyoung Kang, Yoonkyung Park

**Affiliations:** 1 Department of Biomedical Science, Chosun University, Gwangju 61452, Korea; 2 Department of Bioinformatics, Kongju National University, Kongju 38065, Korea

**Keywords:** NF-κB, osteoarthritis, cartilage degeneration, chondrocyte catabolism, chondrocyte apoptosis, IκBζ

## Abstract

Osteoarthritis (OA) is a type of joint disease associated with wear and tear, inflammation, and aging. Mechanical stress along with synovial inflammation promotes the degradation of the extracellular matrix in the cartilage, leading to the breakdown of joint cartilage. The nuclear factor-kappaB (NF-κB) transcription factor has long been recognized as a disease-contributing factor and, thus, has become a therapeutic target for OA. Because NF-κB is a versatile and multi-functional transcription factor involved in various biological processes, a comprehensive understanding of the functions or regulation of NF-κB in the OA pathology will aid in the development of targeted therapeutic strategies to protect the cartilage from OA damage and reduce the risk of potential side-effects. In this review, we discuss the roles of NF-κB in OA chondrocytes and related signaling pathways, including recent findings, to better understand pathological cartilage remodeling and provide potential therapeutic targets that can interfere with NF-κB signaling for OA treatment.

## 1. Introduction

Osteoarthritis (OA) is the most common form of arthritis and is a leading cause of disability that reduces the quality of life and causes economic loss [1]. It occurs when cartilage breaks down and allows bones to rub against each other. The prevalence of OA is continuously increasing because of the rise in the population age and obesity [2,3,4]. However, most pharmacologic therapies for OA such as the oral administration of non-steroidal anti-inflammatory drugs (NSAIDs) and glucosamine are limited to pain management rather than preventions and cures, and surgery is typically a last resort for treating knee OA [5]. Indeed, no licensed disease-modifying drugs are currently available [6], although many clinical trials using the intra-articular (IA) delivery method have been conducted, including treatments with hyaluronic acid, glucocorticoids, biologic agents targeting pro-inflammatory cytokines, and cell therapies using tissue explants, cell concentrates, or mesenchymal stem cells [7,8]. The lack of disease-modifying drugs is, in part, attributable to the incomplete understanding of the mechanisms of OA pathogenesis. Thus, defining the risk factors that cause OA initiation and progression may reveal biomarkers and therapeutic targets for this disease.

Mechanical stresses and elevated pro-inflammatory cytokines in OA joints play causative roles in disrupting cartilage homeostasis [9,10,11]. Studies have identified the nuclear factor-kappaB (NF-κB) transcription factor as abnormally activated in OA and as a disease-contributing factor [12,13,14]. NF-κB participates in many OA-associated events, including chondrocyte catabolism, chondrocyte survival, and synovial inflammation. Thus, NF-κB, its upstream regulators, co-factors, and downstream effectors are regarded as potential targets for the therapeutic intervention of OA [12,15]. We recently found that NF-κB activation requires IkappaB-zeta (IκBζ) in OA chondrocytes, indicating that IκBζ is a potential therapeutic target for OA-associated NF-κB inhibition [16].

Here, we review the literature describing how NF-κB is involved in OA pathophysiology and articular cartilage homeostasis. We provide an overview of NF-κB signaling in OA disease, emphasizing its cartilage catabolism-promoting role. This review also discusses recent findings related to OA-associated NF-κB signaling, including those regarding the IκBζ protein.

## 2. OA Pathogenesis

OA is not a single disease condition, but rather a complex disorder associated with a variety of risk factors that contribute to OA progression. Chronic mechanical stresses, such as joint injury, overload, or overuse, lead to alterations in the articular cartilage, synovium, and bone in OA, such as cartilage degeneration, synovial inflammation, subchondral bone sclerosis, and osteophyte formation [17,18,19].

Articular cartilage is a highly specialized connective tissue in the joints that consists of chondrocytes and the extracellular matrix (ECM) produced by them. The natural cartilage matrix is mainly composed of type-II collagen and aggrecan, providing cartilage with a shock-absorbing capacity [20,21]. Because articular cartilage not only lacks blood vessels or nerves but also has a limited capacity for intrinsic repair, the preservation of chondrocytes in cartilage is paramount to joint health. Chondrocytes maintain cartilage homeostasis by synthesizing ECM, thus preserving the structural and functional integrity of the cartilage. However, in response to OA stimuli, chondrocytes lose their ability to maintain cartilage integrity and their survival. Further, they converse to catabolic cells that secrete matrix-degrading enzymes, such as matrix metalloproteinases (MMPs) and a disintegrin and metalloproteinase with thrombospondin motifs (ADAMTSs) [10], including essential catabolic MMP13 and ADAMTS5 [22,23,24,25]. As a result, catabolic-degrading effects overwhelm the anabolic-protective function in OA chondrocytes, ultimately leading to cartilage degeneration [26]. Chondrocyte catabolism can be stimulated by soluble pro-inflammatory cytokines including interleukin (IL)-1β, tumor necrosis factor (TNF)-α, and IL-6, which are derived from the inflamed OA synovium and damaged cartilage through paracrine or autocrine mechanisms [27,28,29]. NF-κB activated by these inflammatory cytokines and excessive mechanical stresses or ECM degradation products not only induce catabolic gene transcription, but also stimulate inflammatory mediators such as IL-1β, TNF-α, and IL-6 through a positive feedback loop [30,31,32].

Recent studies demonstrated that inflammation in the synovium is one of the key factors leading to OA progression [33,34,35,36,37]. The synovium is a soft connective tissue membrane consisting of layers of fibroblast-like synoviocytes (FLS) lining the space between the joint capsule and joint cavity. The synovial membrane not only provides structural support, but also secretes synovial fluid, which has a lubricating function to reduce joint cartilage friction during movement and supply necessary nutrients to the surrounding cartilage. In damaged joints, degradation products from the cartilage/meniscus and secreted inflammatory factors from chondrocytes stimulate synovial inflammation in OA [27]. FLS play an important role in synovitis by producing inflammatory cytokines that mediate leukocyte recruitment. Many types of infiltrating immune cells, including macrophages, T cells, mast cells, and B cells, as well as their cytokines, including IL-1β, TNFα, and IL-6, are present at higher levels in the OA synovium than in the normal synovium [28], although the overall number of immune cells in the OA synovium is lower than that in the rheumatoid arthritis (RA) synovium [34]. Because NF-κB acts as a general and essential inflammatory mediator in various cell types [38], it also plays a pivotal role in OA synovitis [30,39,40,41,42,43]. In response to joint damage, synovial cells stimulated by inflammatory cytokines and matrix degradation products potentiate NF-κB-dependent signaling pathways, further providing inflammatory mediators that accelerate cartilage destruction. Because the roles of NF-κB in OA synovitis have been well-documented in previous reviews, this review focuses on its role in OA chondrocytes and cartilage.

## 3. General Function and Regulation of NF-κB

NF-κB is an inducible transcription factor with a central role in immune responses, inflammatory responses, cellular differentiation, and the survival of normal and malignant cells [44]. Because NF-κB is involved in so many biological processes, dysregulation of NF-κB pathways is frequently observed in many diseases, such as arthritis, cancer, and autoimmune diseases [45,46,47]. In mammals, NF-κB is composed of homo- and heterodimers of five members of the Rel family, including NF-κB1 (p105/p50), NF-κB2 (p100/p52), RelA (p65), RelB, and c-Rel. The NF-κB signaling system consists of up to 15 different cell type- and stimulus-specific dimer combinations [48,49]. Structurally, transactivation domains are limited in RelA, RelB, and c-Rel and, therefore, homo- and heterodimers between p50 and p52 cannot function as transcription activators [48]. Among the NF-κB dimers, the p65/p50 heterodimer is the prototype. This complex is found in most cell types and acts as a potent transcription factor.

In unstimulated cells, the NF-κB dimers are retained in the cytoplasm through their interaction with inhibitory IκB proteins. Following stimulation, IκB is phosphorylated by IκB kinases (IKKs) and degraded by the proteasome, allowing free NF-κB complexes to translocate to the nucleus, bind to NF-κB response elements, and transactivate the expression of hundreds of immunomodulatory proteins, pro-inflammatory cytokines, chemokines, adhesion molecules, and growth factors [50,51]. NF-κB also induces IκBα, which suppresses NF-κB through a negative feedback mechanism. In addition to dynamic subcellular translocation, NF-κB activity is modulated by its post-translational modifications, such as phosphorylation, acetylation, methylation, and ubiquitination [52]. For example, phosphorylation of p65 at serine 276 leads to the acetylation of lysine 310, which increases the transcriptional activity of NF-κB [53,54]. B-cell lymphoma 3 (Bcl-3) and IκBζ, two atypical members of the IκB family, are also involved in regulating NF-κB. Unlike classic IκB proteins, they associate with p50 or p52 in the nucleus and selectively modulate NF-κB-dependent gene expression [55,56,57,58].

Activation of NF-κB is mediated by two well-characterized types of signaling pathways, the canonical and non-canonical pathways. These pathways are mainly activated by pro-inflammatory signals or factors involved in the development, respectively [59]. Although they differ in signaling mechanisms and biological functions, they also participate in an intricate cross-talk that regulates the diverse functions of NF-κB in context-specific responses [59,60]. The canonical pathway involves NF-κB dimers composed of the p65, c-Rel, and p50 subunits and requires the IKK complex (IKKα/β/γ). This pathway is fast-acting and reversible because of the IκB-dependent negative feedback mechanism. In contrast, the non-canonical pathway predominantly activates p52 and RelB through IKKα [59]. Compared to the canonical pathway, NF-κB activation in the non-canonical pathway is slower and longer-lasting. The canonical p65/p50 complex was found to be crucial for embryonic development and immune system function based on gene knockout (KO) studies [61]. p65 KO mice die at approximately 15–16 days of gestation because of a massive degeneration of the liver due to hepatocyte apoptosis [62]. Mice lacking the p50 subunit show no developmental abnormalities but display various specific immune defects [63].

## 4. Significance of NF-κB in OA Pathogenesis

Disruption of cartilage matrix integrity is caused by enhanced chondrocyte catabolism/apoptosis with reduced chondrocyte anabolism in the articular cartilage [64,65,66]. By using several mouse models of OA [67], genes that either increase or decrease the susceptibility to OA have been identified. One of the better-characterized signaling pathways activated by OA stimuli, such as inflammation and mechanical loading, is the NF-κB pathway [12,13,14]. The significance of NF-κB in OA disease was confirmed through loss-of-function approaches. In cultured chondrocytes, treatment with NF-κB inhibitors reduced IL-1β-induced catabolic gene expression [16,68,69]. In animal models, injury-induced cartilage lesions were alleviated by the knockdown (KD) of NF-κB p65 in the knee joints through IA injection of specific siRNA [11,70]. In this context, reduced concentrations of IL-1β and TNF-α in the synovial fluid of OA are also observed [11]. Not surprisingly, IKKs, as upstream regulators of the NF-κB-activating machinery, have also been implicated in chondrocyte catabolism and cartilage degeneration [43]. For example, IA injection of BMS-345541, a selective inhibitor of IKKα/β, not only prevented the induction of MMP13 and ADAMTS5 at 2 weeks after surgical induction of OA, but also alleviated cartilage lesions at 8 weeks [71].

## 5. Chondrocyte Catabolism Regulated by NF-κB

### 5.1. The Regulation of Matrix-Degrading Enzymes by NF-κB

Understanding the molecular mechanisms by which activated NF-κB turns on cartilage catabolic pathways may provide an insight into potential therapeutic targets. NF-κB directly or indirectly induces the expression of matrix-degrading enzymes and other OA-associated factors, thereby coordinating abnormal cartilage catabolic pathways. NF-κB induces catabolic gene expression through NF-κB response elements located in the promoters of the MMP1, MMP9, and ADAMTS5 genes [72,73,74,75,76,77], as well as promoting the expression of major pro-inflammatory and destructive mediators of OA, including cyclooxygenase 2 (COX2), prostaglandin E2 (PGE2), and inducible nitric oxide synthase (iNOS) [78,79,80,81,82,83]. Particularly, the loss of iNOS appeared to attenuate cartilage destruction in experimental OA [84,85,86]. NF-κB is also capable of up-regulating other transcription factors, such as hypoxia-inducible factor-2α (HIF-2α), ETS domain-containing protein-1 (ELK1), and E74-like factor 3 (ELF3), which, in turn, perpetuates OA disease by modulating inflammatory and catabolic mediators [87,88,89,90,91]. Activated HIF-2α promotes the expression of matrix-degrading enzymes by binding to the HIF-2α-binding motif located in the promoters of catabolic genes [87,88,92]. Moreover, CCAAT/enhancer-binding protein-β (C/EBPβ), a HIF-2α target gene, exacerbates OA progression by directly inducing MMP13 expression [93]. ELK1 directly increases MMP13 in the basic fibroblast growth factor (bFGF)-treated chondrocytes [94]. ELF3, which acts as a downstream target of NF-κB and a co-factor as well as an activator of NF-κB signaling, drives the expression of genes, such as COX2, iNOS, and MMP13 [90,95,96,97]. In a study of murine destabilization of the medial meniscus (DMM) surgery-induced osteoarthritis, genetic ablation of ELF3 in chondrocytes ameliorated OA development and suppressed iNOS and MMP13 expression [98].

Features in OA cartilage, such as the increased expression of matrix-degrading enzymes, are known to resemble the process of endochondral ossification during normal bone formation and growth [99,100]. These chondrocyte hypertrophy-like changes in OA play a role in both initiating and perpetuating OA disease [101]. The expression of chondrocyte hypertrophy markers, such as MMP13, COL10A1, and VEGF, was up-regulated in OA [102,103,104,105]. The role of NF-κB in chondrocyte hypertrophy has been extensively described elsewhere [12,106]. Briefly, NF-κB regulates chondrocyte hypertrophy mainly through SRY-box transcription factor 9 (SOX9), bone morphogenetic protein 2 (BMP2), and HIF-2α. For example, HIF-2α is not only required for hypertrophic differentiation of chondrocytes, but also potently induces the promoter activities of MMP13, COL10A1, and VEGF by binding to hypoxia-responsive elements [87]. Collectively, these studies highlight that NF-κB orchestrates gene expression programs, leading to the production of matrix-degrading enzymes, pro-inflammatory cytokines, and inflammatory mediators by coordinating multilayered signaling networks, thereby contributing to OA onset and development.

### 5.2. Factors That Regulate NF-κB Activity via Direct Interaction

**Figure 1:**
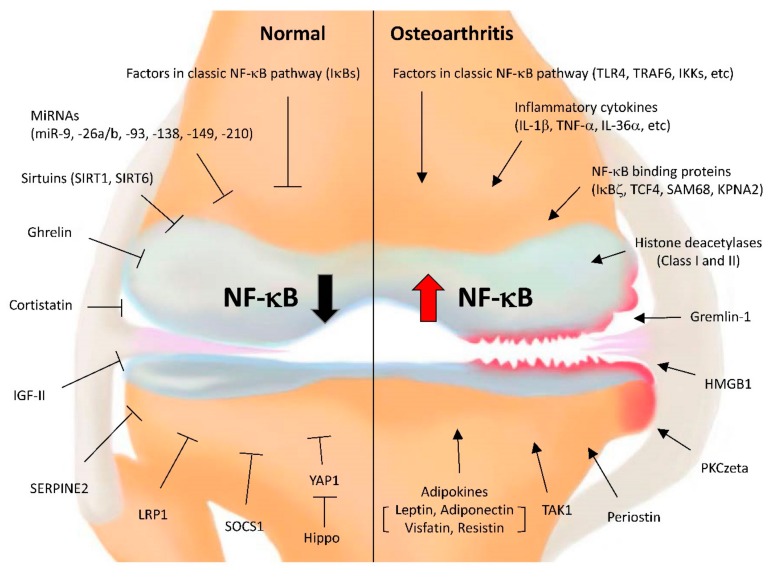
The genes or signaling pathways that positively or negatively regulate NF-κB activation in chondrocytes. In normal chondrocytes, several factors contribute to cartilage homeostasis by maintaining NF-κB activity at a basal level (**left**). In response to OA stimuli, the factors indicated stimulate NF-κB activation, leading to the induction of matrix-degrading enzymes and inflammatory or destructive mediators, eventually causing cartilage destruction (**right**).

The catabolic effects of NF-κB in chondrocytes are potentiated by several NF-κB-binding proteins. Figure 1 lists genes with either stimulatory or inhibitory roles in NF-κB activation in OA chondrocytes. The stimulatory factors that bind to NF-κB subunits include IκBζ, transcription factor 4 (TCF4), SRC-associated in mitosis of 68 kDa (SAM68), and karyopherin alpha 2 (KPNA2). Compared to healthy cartilage, OA cartilage over-expresses all of these proteins [16,107,108,109]. Recently, we showed that IκBζ inhibition may be an alternative therapeutic approach for NF-κB inhibition in OA. IκBζ, an atypical IκB family member, is rapidly induced by NF-κB which, in turn, acts as a transcriptional coactivator of NF-κB in immune cells [55,110]. In chondrocytes, elevated IκBζ forms a complex with the NF-κB p65, p50, and p52 subunits in response to IL-1β and strongly augments NF-κB-dependent transcriptional responses including catabolic genes [16]. Detailed analyses revealed that inactivation of IκBζ in chondrocytes alleviates DMM surgery-induced cartilage destruction but has little effect on synovial inflammation. Thus, IκBζ appears to be necessary for NF-κB to properly activate the gene transcriptional program in OA chondrocytes.

TCF4 is a downstream effector of the Wnt signaling pathway [111]. The overexpression of TCF4 in chondrocytes induced the expression of MMPs and the activation of NF-κB by directly binding to NF-κB p65 and thus competing with IκBα, an endogenous inhibitor of NF-κB [107]. The RNA-binding protein SAM68 can regulate NF-κB activity in several cell types [112,113]. In TNF-α-treated chondrocytes, SAM68 mediates the activation of NF-κB and the expression of catabolic genes [108]. Although a physical interaction between SAM68 and NF-κB p65 was observed, the molecular mechanism of how this binding promotes NF-κB activation remains unclear. KPNA2, a member of the importin α family, modulates p65 nuclear translocation [114]. In IL-1α-treated chondrocytes, KPNA2 promoted p65 nuclear transportation and thus accelerated chondrocyte catabolism [109]. Therefore, interfering with NF-κB signaling by targeting these factors may harbor valuable opportunities for OA treatment.

### 5.3. Factors That Activate NF-κB under OA Conditions

The results of in vitro and in vivo studies demonstrated that increased NF-κB activity through diverse signaling pathways is positively correlated with enhanced cartilage destruction. For instance, the MAP3-kinase TGF-β-activated kinase 1 (TAK1) which links MAP kinase signaling to NF-κB activation has been implicated in OA pathogenesis [115,116]. While IA injection of the TAK1 inhibitor 5Z-7 resulted in reduced NF-κB activation, catabolic factor expression, and OA development in a rat DMM model; IA injection of TAK1-encoding adenovirus caused OA-like cartilage lesions [115].

Increasing evidence has revealed abnormal accumulation of secreted proteins or peptides in the synovial fluid and articular cartilage from OA joints. Secreted proteins may be more efficient therapeutic targets and function as biomarkers. Very recently, Chang et al. identified gremlin-1, an extracellular antagonist of the bone morphogenetic proteins (BMPs), as a critical regulator of excessive mechanical loading-induced OA development [117]. Excessive mechanical loading was previously shown to cause NF-κB activation and OA development, whereas physiological loading protects against cartilage loss and inhibits NF-κB activation at multiple levels including by suppressing TAK1 and IKKβ [13,118,119,120]. While increased levels of gremlin-1 in mouse knee joints led to OA-like phenotypes, inactivation of gremlin-1 in chondrocytes suppressed both post-traumatic and spontaneous OA [117]. The authors suggested that the RAC1-ROS-NF-κB pathway activated by excessive mechanical loading induces gremlin-1, and the secreted gremlin-1 further activates NF-κB-dependent chondrocyte catabolism and suppresses BMPs-dependent anabolism.

Extracellular factors associated with adipokine function, energy homeostasis, and adipose tissue inflammation also play critical roles in OA pathology [121]. Many adipokines, such as leptin, adiponectin, visfatin, and resistin, have stimulatory effects on cartilage destruction. These adipokines are not only up-regulated in OA but also induce the expression of matrix-degrading enzymes and/or pro-inflammatory mediators in chondrocytes through mechanisms involving NF-κB [122,123,124,125,126,127,128]. This phenomenon is inversely correlated with the effect of ghrelin, a peptide hormone that has the opposite effect to leptin in energy expenditure [129], as ghrelin plays a protective role in DMM-induced OA and IL-1β-induced NF-κB activation [130]. Osteopontin (OPN) and periostin (osteoblast-specific factor 2; OSF2), which are secreted factors involved in adipose tissue inflammation and bone remodeling [131,132,133,134,135], have also been implicated in OA. The OPN level is higher in the OA synovial fluid and articular cartilage of patients with OA [136,137]. OPN promotes MMP13 expression through NF-κB activation in chondrocytes [138]. However, it was also reported that OPN can suppress HIF-2α expression in chondrocytes [139]. Moreover, OPN KO mice exhibited enhanced OA progression induced by both aging and instability [140]. Considering these contradictory findings, detailed analyses may be necessary to clarify these issues. Like OPN, periostin is up-regulated in human OA cartilage, and treatment with periostin in human chondrocytes activates the NF-κB-dependent induction of catabolic genes and inflammatory cytokines [141].

Inflammatory cytokines derived from the OA synovium or damaged cartilage are well-known to cause catabolic gene induction in chondrocytes through mechanisms involving NF-κB activation [142,143]. Thus, treatment of chondrocytes with traditional inflammatory cytokines, such as IL-1β and TNF-α, has been widely used to prepare in vitro OA models. IL-6, a well-known NF-κB target gene, also has a causative role in OA progression [82]. Recently, IL-36α, a member of the IL-1 cytokine subfamily, was suggested as a potent OA-inducing factor. IL-36α is highly expressed in inflamed joints [144]. Conde et al. observed the up-regulation of IL-36α in OA cartilage compared to in healthy cartilage and showed that IL-36α has catabolic roles in chondrocytes by activating NF-κB [145]. Very recently, the TGF-β-IL-36α axis was proposed as a critical signaling pathway in OA pathology [146]. In normal joints, TGF-β signaling plays a protective role in maintaining chondrocytes [147,148,149,150]. Li et al. found that inactivation of TGF-β type 2 receptor (TGFBR2) by joint damage triggers the induction of IL-36α, leading to NF-κB- and the MAPK-dependent activation of MMP13 and eventually causing OA cartilage destruction [146]. This study also revealed an endogenous IL-36 receptor antagonist as a potential therapeutic target. Another inflammatory mediator, high mobility group box 1 (HMGB1), which is a chromatin protein with a dual function as a nuclear factor and extracellular factor, can also activate the NF-κB signaling pathway in chondrocytes. In addition to its primary roles in RA and OA synovitis as an inflammatory mediator [151,152,153], HMGB1 can regulate the IL-1β-induced activation of NF-κB and expression of catabolic genes in chondrocytes [154].

Several pharmacologic inhibitors have been shown to be effective in in vitro or in vivo OA models, with some executing their functions through NF-κB inhibition. The inhibitors reported have been developed against protein kinase Czeta (PKCzeta) [155,156], purinergic P2X7 receptor (P2X7R) [157], specificity protein 1 (SP1) [158], and receptor-interacting protein kinase 1 (RIPK1) [159]. Similar to the anti-catabolic effects of the pharmacologic PKCzeta inhibitor, inhibition of PKCzeta using molecular approaches, such as siRNA-mediated KD and the overexpression of dominant negative PKCzeta, also suppressed ECM degradation in inflammatory cytokine-treated chondrocytes [155,156]. In contrast, the SP1 inhibitor mithramycin A showed anti-catabolic effects in chondrocytes by primarily suppressing the NF-κB-HIF2-α axis rather than by targeting SP1, as SP1 KD had minimal effects on catabolic gene expression in IL-1β-treated chondrocytes [158]. Collectively, these studies identified potential signaling pathways and targets for NF-κB inhibition.

### 5.4. Factors That Inhibit NF-κB in OA Conditions

Reduced OA severity is often observed when NF-κB is restrained by factors required to maintain cartilage homeostasis. These inhibitory factors that reduce NF-κB activity in chondrocyte catabolism include yes-associated protein 1 (YAP1), cortistatin (CST), insulin-like growth factor II (IGF-II), serpin family E member 2 (SERPINE2), low-density lipoprotein receptor-related protein 1 (LRP1), and cytokine signaling-1 suppressor (SOCS1). Recently, Deng et al. identified YAP1 as a critical negative regulator of NF-κB activity in OA [160]. Mechanistically, inflammatory cytokines induce Hippo signaling activation and TAK1-dependent degradation of YAP1, leading to IKKα/β-NF-κB cascade activation. The authors also showed that cartilage-specific KO of YAP1 exaggerates experimental OA by enhancing chondrocyte catabolism. Further, OA development was alleviated by depletion of MST1/2, which are upstream inhibitory kinases of YAP1 in the Hippo signaling pathway. CST (a neuropeptide) inhibits NF-κB activation in chondrocytes by antagonizing TNF-α function via direct binding to TNF receptors [161]. Studies in both spontaneous and surgically induced OA models indicated that the CST deficiency leads to an accelerated OA-like phenotype, while exogenous CST attenuates OA development in vivo. IGF-II, an insulin-like growth factor, was found to be down-regulated in human OA cartilage [162]. Overexpression of IGF-II in chondrocytes or mouse knee joints decreased IL-1β-induced NF-κB activation or experimental OA progression. Studies of SERPINE2, LRP1, and SOCS1 are limited to in vitro chondrocytes, but these proteins were shown to negatively regulate inflammatory cytokine-induced activation of NF-κB and the expression of catabolic factors in chondrocytes [163,164,165]. These inhibitory factors may be useful for overcoming NF-κB activation in OA cartilage destruction.

## 6. Epigenetics Associated with NF-κB in OA

### 6.1. Histone Deacetylases (HDACs)

Epigenetic alterations in histone and non-histone proteins occur in OA disease [166,167]. In fact, HDACs appeared to affect NF-κB activity and catabolic gene expression in chondrocytes. Interestingly, the opposite effects on OA pathology were observed following inhibition of NAD-dependent deacetylases (Class III) or the classical zinc-dependent histone deacetylases (Class I and II). Class III HDACs, the sirtuins (SIRT1-7), share a common catalytic core domain. Among them, SIRT1 and SIRT6 were found to inhibit NF-κB p65 activity via direct deacetylation of the NF-κB p65 subunit at lysine 310 (SIRT1) [168,169,170] or deacetylation of histone H3 on NF-κB target gene promoters (SIRT6) [171]. In joints, treatment with SIRT1 activators or overexpression of SIRT6 attenuated experimental OA progression and suppressed pro-inflammatory cytokine-induced catabolic gene expression in chondrocytes, whereas cartilage-specific KO of SIRT1 accelerated OA development [172,173,174,175,176]. These reports indicate the overall protective effects of SIRT1 and SIRT6 in maintaining cartilage integrity. In support of this concept, the inhibition of the cytosolic acetyl-CoA biosynthesis pathway inhibited IL-1β-induced acetylation of p65 and catabolic gene expression [177]. SIRT2, the closest homolog of SIRT1, can deacetylate p65 at K310 [178], but its role in OA pathogenesis remains unclear.

In contrast, the classical histone deacetylases (Class I and II) promote OA development. Treatment with a pan-HDAC inhibitor (SAHA) that inhibits HDAC1-10 or an HDAC6-specific inhibitor (ACY-1215) inhibited NF-κB activation and catabolic gene expression in IL-1β-stimulated chondrocytes [179,180]. Another pan-HDAC inhibitor, trichostatin A (TSA), also alleviated experimental OA progression [181,182]. Although further studies are required to define the detailed molecular mechanism including discrimination of deacetylase isoforms, these studies indicate that the activation of sirtuins or the inhibition of classical HDACs has beneficial effects in the management of OA conditions.

### 6.2. MicroRNAs

MicroRNAs (miRNAs) participate in OA pathogenesis [183,184,185]; here, we summarize the miRNAs associated with NF-κB signaling in chondrocytes (Table 1). MiRNAs that are positively regulated by NF-κB signaling include miR-27b [186], miR-140 [187], miR-146a [188,189], miR-204 [190], and miR-365 [191], whereas the miRNAs down-regulated by NF-κB are miR-26a-5p [192], miR-92a-3p [193], miR-320 [194], and miR-558 [195]. These previous studies indicated that the NF-κB signaling network is an important component of miRNA signaling in OA.

Several miRNAs induced by NF-κB promote OA cartilage destruction. For instance, miR-365 is up-regulated in the knee joints of OA patients and promotes catabolic factor expression by targeting HDAC4 [191]. Very recently, Kang et al. identified a critical miRNA for OA pathogenesis [190]. MiR-204 induced by NF-κB in response to senescence stimuli facilitates OA cartilage destruction by targeting multiple components of the proteoglycan biosynthesis pathway.

In contrast, increasing evidence has shown that many types of miRNAs suppress chondrocyte catabolism by inhibiting matrix-degrading enzymes or molecular components of the NF-κB signaling pathway. Specifically, miR-27b, miR-140, and miR-320 target MMP13 [186,187,194], whereas miR-92a-3p inhibits ADAMTS4/5 [193]. MiR-138 and miR-9 were suggested to directly suppress the NF-κB subunits p65 or p105/50 [14,196]. Several miRNAs have the potential to suppress NF-κB signaling by targeting upstream regulators of NF-κB, such as Toll-like receptor 4 (TLR4) (by miR-93) [197], death receptor 6 (DR6) (by miR-210) [198], KPNA3 (by miR-26a/b) [199], TAK1 (by miR-149) [200], and TNF-receptor associated factor 6 (TRAF6)/interleukin-1 receptor associated kinase 1 (IRAK1) (by miR-146a) [188,201]. MiR-558 and miR-26-5p target NF-κB-downstream COX2 and iNOS, respectively [192,195]. With regard to miR-146a, contradictory conclusions regarding its roles in OA have been reported. While miR-146a overexpression can decrease catabolic factor expressions by targeting TRAF6 in chondrocytes and nucleus pulposus cells from the intervertebral disc [202,203], other reports showed that miR-146a may promote OA pathogenesis by disrupting TGF-β signaling through the targeting of Smad4 and by increasing apoptosis [204,205]. In support of the protective role of miR-146a in OA, one of the above-mentioned groups recently reported that either the genetic deletion of miR-146a or IA treatment with miR-146a inhibitor alleviated cartilage lesions induced by DMM surgery [206].

## 7. Chondrocyte Apoptosis Regulated by NF-κB

Dysregulation of chondrocyte survival may lead to ECM loss and cartilage destruction, as chondrocytes are the only cell type present in the cartilage. Many studies have demonstrated correlations between chondrocyte apoptosis and OA severity [207,208,209,210], and IA injection with a caspase inhibitor reduced cartilage lesions in a rabbit ACLT transection model of OA [211]. Although it remains unclear whether chondrocyte apoptosis is the inducer of cartilage degeneration or a byproduct of cartilage destruction [64,212], chondrocyte apoptosis is an important aspect of OA pathogenesis.

NF-κB has biphasic roles in chondrocyte survival and apoptosis. NF-κB is known to prevent TNF-α-induced cell death, and this effect is associated with the induction of anti-apoptotic genes [213,214,215]. In chondrocytes, TNF-α-induced apoptosis is also reduced by NF-κB inhibition [216,217]. In support of these findings, Nkx3.2-dependent activation of p65 enhanced chondrocyte survival and reduced apoptosis in ATDC5 cells [218].

In contrast, NF-κB may also have a pro-apoptotic function depending on the stimulus and cellular environment. Several factors that activate NF-κB, such as TCF4 [107], SAM68 [108,112], and RIPK1 [159], were found to potentiate apoptosis in cultured chondrocytes. Moreover, HIF-2α, an NF-κB target gene, accelerated Fas-mediated chondrocyte apoptosis in OA cartilage [219]. MiR-9, which targets the NF-κB p50 subunit, promoted chondrocyte survival [14]. Ding et al. also found that miR-93, which inhibits the NF-κB pathway by targeting TLR4, can suppress chondrocyte apoptosis in lipopolysaccharide (LPS)-treated primary chondrocytes and in a medial meniscectomy tear (MMT) surgery model [197]. Recently, Yan et al. supported the pro-apoptotic function of NF-κB, based on the finding that p65 KD in mouse knee joints inhibited early chondrocyte apoptosis caused by joint impact injury [70].

**Figure 2:**
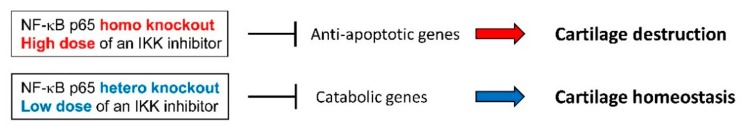
The nuclear factor-kappaB (NF-κB) level differently regulates cartilage homeostasis and Osteoarthritis (OA). While the complete inactivation of NF-κB p65 in chondrocytes or a high dose of IκB kinases (IKK) inhibitor causes OA development by inducing chondrocyte apoptosis, p65 hetero knockout or a low dose of IKK inhibitor alleviates OA by suppressing matrix-degrading enzymes.

By using a cartilage-specific p65 KO model in adult mice, Kobayashi et al. showed that the p65 level in chondrocytes determines whether cartilage undergoes homeostasis or destruction (Figure 2) [220]. Specifically, hetero-KO of p65 in adult chondrocytes or a low dose of an IKK inhibitor showed predictable suppressive effects on both OA development and chondrocyte catabolism without affecting cell survival, whereas the complete depletion of p65 using homo-KO accelerated OA by enhancing chondrocyte apoptosis. In support of this finding, the same group later reported that higher doses of IKK inhibitor did not alleviate OA development but rather promoted chondrocyte apoptosis [71]. Considering these reports and the roles of NF-κB in numerous cellular processes, such as cell survival, it may be important to develop strategies for inhibiting OA-responsive NF-κB signaling pathways rather than the physiological p65 function. Notably, homo-KO of IκBζ in chondrocytes did not affect both the normal skeletal development or chondrocyte survival, but significantly suppressed NF-κB-dependent chondrocyte catabolism [16]. Thus, IκBζ inhibition may be a useful therapeutic approach for NF-κB inhibition in OA.

## 8. Conclusions

Until recently, many pharmacologic agents have been tried to treat pain and loss of function associated with OA. Some of these drugs were shown to slow OA progression, but placebo effects and increased side effects were often observed [8]. Clinical trial-based studies using biologic agents targeting specific genes have focused on blocking the inflammatory response within the synovium and articular chondrocytes, but such modalities did not show promising effects in OA treatment [8,221]. NF-κB signaling pathways provide multiple avenues for targeting OA because many OA-causing signaling pathways are known to be interconnected by NF-κB. Therefore, in this review, we summarized the published results regarding the significance of NF-κB and its regulation in OA cartilage. Cross-talk between NF-κB and newly recognized genes or pathways may reveal numerous potential targets for pharmacological treatment to slow or reverse OA progression. However, how to selectively inhibit OA-specific functions of NF-κB rather than physiological responses is important and further studies are required to avoid unwanted side effects of this non-life-threatening disease. Therefore, more detailed knowledge of NF-κB and its signaling pathways is needed to better understand the clinical features of OA and for translational studies.

## Figures and Tables

**Table 1:** The microRNAs involved in nuclear factor-kappaB (NF-κB) signaling in Osteoarthritis (OA) chondrocytes.

miRNA(s)	Regulation by NF-κB	Target Gene(s)	Function(s) in Chondrocytes	Reference
miR-365	Increased	HDAC4	Promotes catabolism	[191]
miR-204	Increased	Multiple genes in PG biosynthesis pathway	Promotes OA development	[190]
miR-27b,-140	Increased	MMP13	Inhibits catabolism	[186,187]
miR-320	Decreased	MMP13	Inhibits catabolism	[194]
miR-92a-3p	Decreased	ADAMTS4/5	Inhibits catabolism	[193]
miR-9	ND	NF-κB p105/50	Directly inhibits NF-κB	[14]
miR-138	ND	NF-κB p65	Directly inhibits NF-κB	[196]
miR-93	ND	TLR4	Inhibits NF-κB upstream	[197]
miR-210	ND	DR6	Inhibits NF-κB upstream	[198]
miR-26a/b	ND	KPNA3	Inhibits NF-κB upstream	[199]
miR-149	ND	TAK1	Inhibits NF-κB upstream	[200]
miR-146a	Increased	TRAF6/IRAK1	Inhibits NF-κB upstream	[189,201]
		Smad4	Promotes OA development	[204,205,206]
miR-26a-5p	Decreased	iNOS	Inhibits NF-κB downstream	[192]
miR-558	Decreased	COX2	Inhibits NF-κB downstream	[195]

Abbreviations: miRNA, microRNA; PG, proteoglycan; OA, osteoarthritis; ND, not determined.
